# Pharmacokinetics of Temsavir, the Active Moiety of the HIV-1 Attachment Inhibitor Prodrug, Fostemsavir, Coadministered with Cobicistat, Etravirine, Darunavir/Cobicistat, or Darunavir/Ritonavir with or without Etravirine in Healthy Participants

**DOI:** 10.1128/aac.02251-21

**Published:** 2022-03-22

**Authors:** Katy Moore, Nilay Thakkar, Mindy Magee, Heather Sevinsky, Blisse Vakkalagadda, Susan Lubin, Cyril Llamoso, Peter Ackerman

**Affiliations:** a ViiV Healthcare, Research Triangle Park, North Carolina, USA; b GlaxoSmithKline, Upper Providence, Pennsylvania, USA; c Bristol-Myers Squibb, Hopewell, New Jersey, USA; d ViiV Healthcare, Branford, Connecticut, USA

**Keywords:** antiretroviral agents, BCRP, CYP3A4, drug-drug interaction, exposure, fostemsavir, heavily treatment experienced, P-glycoprotein

## Abstract

Fostemsavir is a prodrug of temsavir, a first-in-class attachment inhibitor that binds directly to HIV-1 gp120, preventing initial viral attachment and entry into host CD4^+^ T cells with demonstrated efficacy in phase 2 and 3. Temsavir is a P-glycoprotein and breast cancer resistance protein (BCRP) substrate; its metabolism is mediated by esterase and CYP3A4 enzymes. Drugs that induce or inhibit CYP3A, P-glycoprotein, and BCRP may affect temsavir concentrations. Understanding potential drug-drug interactions (DDIs) following fostemsavir coadministration with antiretrovirals approved for HIV-1-infected treatment-experienced patients, including darunavir plus cobicistat (DRV/c) or DRV plus low-dose ritonavir (DRV/r) and etravirine, is clinically relevant. Open-label, single-sequence, multiple-dose, multicohort DDI studies were conducted in healthy participants (*n* = 46; *n* = 32). The primary objective was to assess the effects of DRV/r, etravirine, DRV/r plus etravirine, cobicistat, and DRV/c on temsavir systemic exposures; safety was a secondary objective. Compared with fostemsavir alone, coadministration with DRV/r increased the temsavir maximum observed plasma concentration (*C*_max_), area under the concentration-time curve in one dosing interval (AUC_tau_), and plasma trough concentration (*C*_tau_) by 52%, 63%, and 88%, respectively, while etravirine decreased the temsavir *C*_max_, AUC_tau_, and *C*_tau_ by ∼50% each. DRV/r plus etravirine increased the temsavir *C*_max_, AUC_tau_, and *C*_tau_ by 53%, 34%, and 33%, respectively. Compared with fostemsavir alone, coadministration with cobicistat increased the temsavir *C*_max_, AUC_tau_, and *C*_tau_ by 71%, 93%, and 136%, respectively; DRV/c increased the temsavir *C*_max_, AUC_tau_, and *C*_tau_ by 79%, 97%, and 124%, respectively. Fostemsavir with all combinations was generally well tolerated. No dose adjustment is required for fostemsavir when coadministered with strong CYP3A inhibitors, P-glycoprotein inhibitors, and modest inducers, including regimens with DRV/r, DRV/c, cobicistat, etravirine, and DRV/r plus etravirine based on the therapeutic margin for temsavir (ClinicalTrials.gov registration no. NCT02063360 and NCT02277600).

## INTRODUCTION

In the absence of a cure for HIV-1 infection, there is a continued need for new antiretroviral (ARV) agents to provide viable options for individuals whose treatment choices are limited by drug resistance, intolerability, or drug-drug interactions (DDIs) (1–3). Therefore, it is important that new ARVs with novel mechanisms of action are not only active against drug-resistant strains and tolerable, but also have a DDI profile making them amenable for effective use in combination with other ARVs and concomitant medications.

Fostemsavir (FTR) is an oral prodrug that is metabolized to the active moiety temsavir (TMR), a first-in-class, HIV-1 attachment inhibitor. TMR prevents the initial interaction between the virus and host cellular CD4^+^ immune cells by specifically binding to the viral envelope protein gp120 near the CD4 receptor site (4). This binding prevents the interaction of the virus and host CD4^+^ cells, blocking viral entry and stopping the first step in the HIV replication life cycle (4). FTR has a unique resistance profile with no *in vitro* cross-resistance observed with other classes of ARVs, including entry inhibitors, and can be used regardless of viral tropism (CCR5, CXCR4, or dual-mixed). FTR is administered as an extended-release formulation and is converted via alkaline phosphatase in the gastrointestinal lumen to the highly permeable active moiety TMR (5). Preclinical studies demonstrated that TMR is a P-glycoprotein (P-gp) and breast cancer resistance protein (BCRP) substrate and that its metabolism is mediated by esterase and cytochrome P450 3A (CYP3A4) enzymes (6). Thus, coadministration of FTR with inhibitors or inducers of esterases, CYP3A4, P-gp, and/or BCRP may impact TMR concentrations. A previous study demonstrated that ritonavir (RTV), a strong CYP3A4 and P-gp inhibitor, increased TMR exposures by 44 to 53% (7).

In a phase 2b study (ClinicalTrials.gov registration no. NCT01384734), a 7-day lead-in FTR monotherapy substudy showed that the range of median declines in HIV-1 RNA across the four different FTR treatment groups were 0.69 to 1.4 log_10_ copies/mL (8), and after 96 weeks of combination antiretroviral therapy (cART) with raltegravir (RAL) and tenofovir disoproxil fumarate (TDF), FTR showed similar efficacy to an active atazanavir/ritonavir (ATV/r) reference arm (8, 9). FTR was generally well tolerated through week 96 with an adverse event (AE) profile similar to or more favorable than that of the reference group (10). The phase 3 BRIGHTE study (ClinicalTrials.gov registration no. NCT02362503) conducted in heavily treatment-experienced (HTE) HIV-1-infected participants (defined as those who have ≤2 active ARV classes remaining due to resistance, intolerabilities, and/or contraindications) demonstrated good efficacy (virological and immunological response) and a favorable safety and tolerability profile through 48 weeks of treatment with FTR plus optimized background therapy (11). FTR in combination with a failing regimen achieved its primary endpoint of superior efficacy relative to placebo in HTE, HIV-1-infected participants in the randomized cohort, with an adjusted mean decline of 0.8 log_10_ copies/mL (*P < *0.0001) in HIV-1 RNA through 8 days of FTR functional monotherapy. The safety profile of FTR was consistent across phase 2 and 3 studies.

Darunavir (DRV), a protease inhibitor (PI) routinely boosted with low-dose RTV (DRV/r) or cobicistat (COBI; DRV/c), and etravirine (ETR), a nonnucleoside reverse transcriptase inhibitor, are approved for use in treatment-experienced HIV-1-infected patients (12, 13). COBI and low-dose RTV are both mechanism-based CYP3A inhibitors and are used as pharmacokinetic (PK) enhancers of ARVs, primarily PIs and certain integrase inhibitors (12, 14–17). Both RTV and COBI are inhibitors of the transporters P-gp, OATP1B1, and OATP1B3 (14, 17). In addition, COBI is an inhibitor of BCRP and MATE1 (14); RTV may induce glucuronidation, CYP1A2, CYP2C8, CYP2C9, and CYP2C19 (12), and ETR is an inducer of CYP3A and an inhibitor of CYP2C9, CYP2C19, and P-gp (13).

As HTE HIV-1-infected patients may also be receiving boosted PIs and ETR as part of their ARV regimen, investigating the potential for any DDIs between FTR and these compounds is of clinical relevance. Here, we present two studies—206281, which assessed the effects of DRV/r, ETR, and DRV/r plus ETR on the systemic exposures of TMR, and 206285, which assessed the effects of COBI and DRV/c on the systemic exposures of TMR.

## RESULTS

### 206281 participant disposition/baseline demographics.

Of the 100 individuals who consented to participate, 46 entered the treatment period and were randomly assigned to one of three cohorts; 14 participants each were assigned to cohorts 1 and 2, and 18 to cohort 3. Four additional participants were enrolled in cohort 3 to replace participants who discontinued due to AEs. Baseline demographics were generally balanced between treatment groups; the median age was 33.5 years (range, 23 to 49 years), most participants were male (65.2%) and white (56.5%), and the median body weight was 76.65 kg (53.4 to 109.0 kg) (see Tables S1 and S2 in the supplemental material).

### TMR PK in 206281.

**FIG 1:**
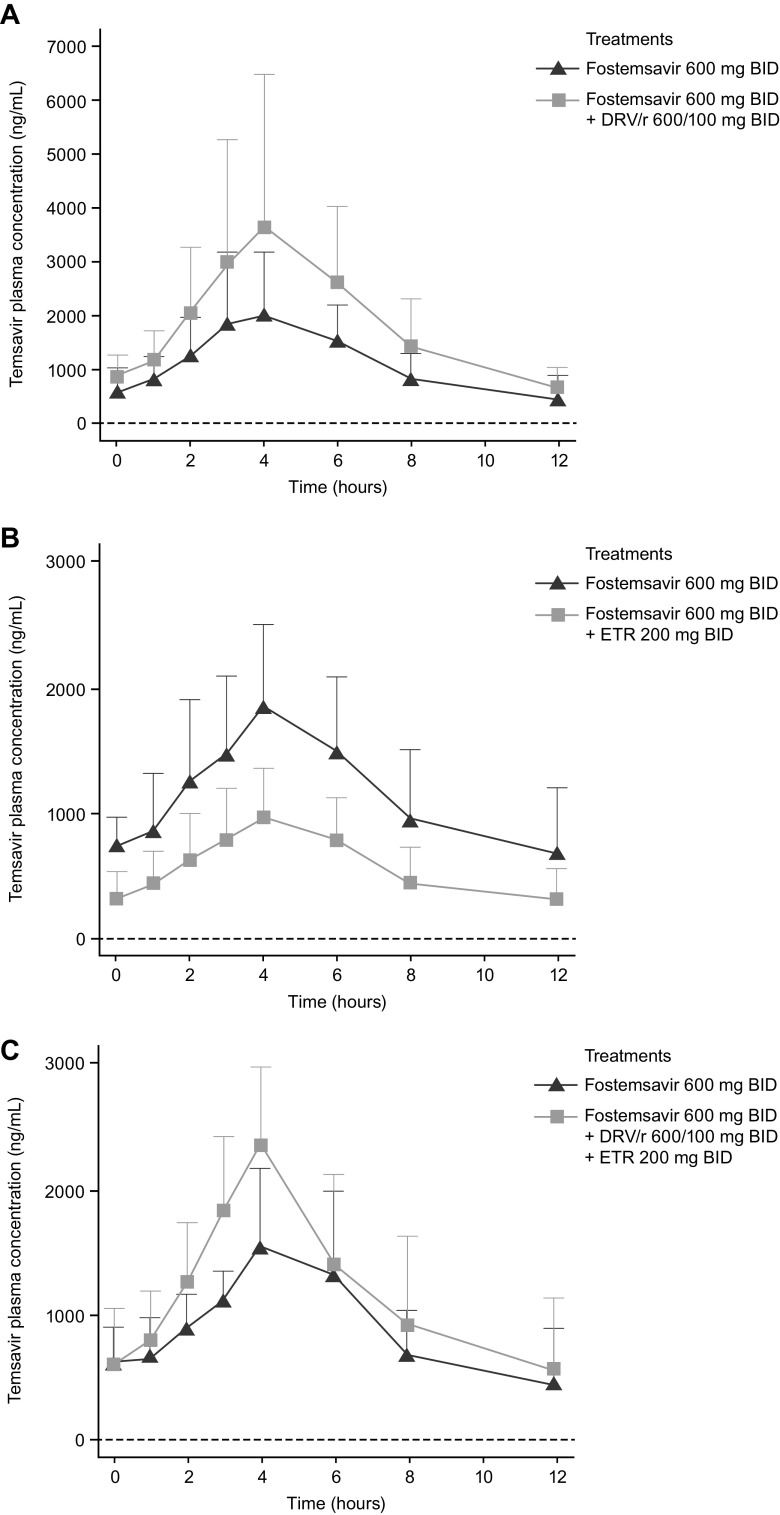
Effect of (A) DRV/r, (B) ETR, and (C) ETR plus DRV/r on the PK parameters of temsavir (206281). BID, twice daily; DRV/r, ritonavir-boosted darunavir; ETR, etravirine; PK, pharmacokinetics.

**(i) Effect of DRV/r on TMR PK.** Compared with FTR alone, coadministration with DRV/r increased the TMR maximum observed plasma concentration (*C*_max_), area under the concentration-time curve in one dosing interval (AUC_tau_), and concentration at 12 h after the last dose (*C*_12_) by 52%, 63%, and 88%, respectively (Fig.1A and Table 1). There was no clinically relevant change in the TMR time of *C*_max_ (*T*_max_; median, minimum to maximum [min-max]) when FTR was coadministered with DRV/r (4.03, 3.00 to 8.00 h) relative to administration of FTR alone (4.00, 2.02 to 6.05 h).

**TABLE 1:** Effect of DRV/r, ETR, and ETR plus DRV/r on PK parameters of temsavir (study 206281)^*a*^

Treatment and comparison	Temsavir PK parameter, adjusted geometric means		
	*C*_max_, ng/mL (90% CI)	AUC_tau_, h · ng/mL (90% CI)	*C*_12_, ng/mL (90% CI)
Effect of DRV/r on PK of temsavir (cohort 1)			
Fostemsavir (*n* = 14)	2,033 (1,619–2,553)	12,632 (10,142–15,735)	308 (213–444)
Fostemsavir + DRV/r (*n* = 12)	3,098 (2,386–4,022)	20,633 (16,333–26,065)	577 (455–732)
GMR for fostemsavir + DRV/r vs fostemsavir	1.524 (1.279–1.815)	1.633 (1.423–1.875)	1.875 (1.093–3.216)
Effect of ETR on PK of temsavir (cohort 2)			
Fostemsavir (*n* = 14)	1,941 (1,699–2,219)	13,364 (11,574–15,430)	479 (310–740)
Fostemsavir + ETR (*n* = 12)	1,003 (858–1,171)	6,714 (5,815–7,753)	231 (156–344)
GMR for fostemsavir + ETR vs fostemsavir	0.516 (0.454–0.587)	0.502 (0.442–0.571)	0.483 (0.324–0.720)
Effect of ETR + DRV/r on PK of temsavir (cohort 3)			
Fostemsavir (*n* = 14)	1,586 (1,362–1,805)	10,339 (9,136–11,700)	312 (221–441)
Fostemsavir + DRV/r + ETR (*n* = 12)	2,398 (2,144–2,682)	13,861 (11,659–16,478)	416 (286–603)
GMR for fostemsavir + DRV/r + ETR vs fostemsavir	1.529 (1.323–1.768)	1.341 (1.172–1.534)	1.332 (0.980–1.809)

a AUC_tau_, area under the concentration-time curve in one dosing interval; *C*_12_, concentration at 12 h after the last dose; CI, confidence interval; *C*_max_, maximum observed plasma concentration; DRV/r, ritonavir-boosted darunavir; ETR, etravirine; GMR, geometric mean ratio; PK, pharmacokinetic.

**(ii) Effect of ETR on TMR PK.** Compared with FTR alone, coadministration with ETR reduced the TMR *C*_max_, AUC_tau_, and *C*_12_ by 48%, 50%, and 52%, respectively (Fig. 1B and Table 1). There was no clinically relevant change in the TMR *T*_max_ (median, min-max) when FTR was coadministered with ETR (4.00, 4.00 to 6.00 h) relative to administration of FTR alone (4.00, 3.00 to 12.0 h).

**(iii) Effect of ETR plus DRV/r on TMR PK.** Compared with FTR alone, coadministration with DRV/r and ETR increased the TMR *C*_max_, AUC_tau_, and *C*_12_ by 53%, 34%, and 33%, respectively (Fig. 1C and Table 1). There was no clinically relevant change in the TMR *T*_max_ (median, min-max) when FTR was coadministered with DRV/r plus ETR (4.00, 3.00 to 8.00 h) relative to administration of FTR alone (4.00, 0.00 to 6.00 h in cohort 3).

### PK of RTV, DRV, and ETR in 206281.

**(i) Effect of FTR on DRV PK.** In cohort 1, coadministration of FTR with DRV/r resulted in no meaningful change for the DRV *C*_max_, AUC_tau_, or *C*_12_. In cohort 3, coadministration of FTR with DRV/r + ETR resulted in 5%, 6%, and 12% decreases, respectively, for the *C*_max_, AUC_tau_, and *C*_12_ of DRV. The 90% confidence intervals (CIs) of DRV ratios of geometric means (GMRs) comparing FTR plus DRV/r plus ETR versus DRV/r plus ETR were contained within the predefined 90% CI limits for DRV of 0.75 to 1.70 for all three parameters in both cohorts (Table 2).

**TABLE 2:** Effect of fostemsavir on PK parameters of RTV, DRV, and ETR^*a*^

Treatment and comparison	PK parameter, adjusted geometric means		
	*C*_max_, ng/mL (90% CI)	AUC_tau_, h · ng/mL (90% CI)	*C*_12_, ng/mL (90% CI)
Effect of fostemsavir coadministration on PK of DRV			
Cohort 1			
DRV/r (*n* = 13)	8,785 (8,181–9,434)	68,380 (63,785–73,306)	3,633 (3,255–4,056)
Fostemsavir + DRV/r (*n* = 12)	8,636 (7,983–9,342)	64,543 (58,485–71,228)	3,445 (2,956–4,016)
GMR of Fostemsavir + DRV/r vs DRV/r	0.983 (0.931–1.038)	0.944 (0.894–0.996)	0.948 (0.865–1.040)
Cohort 3			
DRV/r + ETR (*n* = 13)	10,105 (9,524–10,721)	77,739 (72,510–83,344)	3,672 (3,171–4,252)
Fostemsavir + DRV/r + ETR (*n* = 13)	9,636 (8,920–10,409)	72,907 (66,302–80,171)	3,234 (2,628–3,979)
GMR for fostemsavir + ETR vs fostemsavir + DRV/r + ETR	0.954 (0.903–1.007)	0.938 (0.888–0.991)	0.881 (0.769–1.009)
Effect of fostemsavir on PK parameters of RTV			
Cohort 1			
DRV/r (*n* = 13)	1,324 (1,123–1,560)	7,191 (6,082–8,501)	229 (182–288)
Fostemsavir + DRV/r (*n* = 12)	1,317 (1,050–1,651)	8,256 (6,457–10,557)	273 (204–366)
GMR for fostemsavir + DRV/r vs DRV/r	0.995 (0.856–1.156)	1.148 (0.992–1.329)	1.192 (1.056–1.347)
Cohort 3			
DRV/r + ETR (*n* = 13)	862 (721–1,031)	4,928 (4,323–5,617)	157 (139–178)
Fostemsavir + DRV/r + ETR (*n* = 13)	982 (828–1165)	5,380 (4,912–5,892)	168 (148–191)
GMR for fostemsavir + ETR vs fostemsavir + DRV/r + ETR	1.139 (0.960–1.352)	1.092 (0.979–1.218)	1.067 (0.972–1.171)
Effect of fostemsavir coadministration on PK of ETR			
Cohort 1			
ETR (*n* = 14)	846 (739–968)	7,825 (6,829–8,965)	457 (392–534)
Fostemsavir + ETR (*n* = 14)	943 (811–1,096)	8,670 (7,542–9,968)	522 (448–609)
GMR for fostemsavir + ETR vs ETR	1.114 (1.039–1.194)	1.108 (1.053–1.166)	1.142 (1.079–1.208)
Cohort 3			
DRV/r + ETR (*n* = 13)	838 (672–1,044)	7,102 (5,577–9,045)	417 (306–570)
Fostemsavir + DRV/r + ETR (*n* = 13)	989 (767–1,274)	9,071 (6,790–12,118)	533 (373–762)
GMR for fostemsavir + ETR vs fostemsavir + DRV/r + ETR	1.180 (1.098–1.267)	1.277 (1.198–1.361)	1.278 (1.175–1.389)

a AUC_tau_, area under the concentration-time curve in one dosing interval; *C*_12_, concentration at 12 h after the last dose; CI, confidence interval; *C*_max_, maximum observed plasma concentration; DRV, darunavir; DRV/r, ritonavir-boosted darunavir; ETR, etravirine; GMR, geometric mean ratio; PK, pharmacokinetic; RTV, ritonavir.

**(ii) Effect of FTR on RTV PK.** In cohort 1, coadministration of FTR with DRV/r resulted in no meaningful change for the *C*_max_ and 15% and 19% increases for the AUC_tau_ and *C*_12_, respectively, for RTV. In cohort 3, coadministration of FTR plus DRV/r plus ETR resulted in an increase of 14% for the RTV *C*_max_ and a 9% and 7% increase for the RTV AUC_tau_ and *C*_12_, respectively (Table 3); however, the 90% CIs for the AUC_tau_ and *C*_12_ were contained within 0.80 to 1.25.

**TABLE 3:** Effect of DRV/c and COBI alone on the PK parameters of temsavir (study 206285)^*a*^

Treatment and comparison	Temsavir PK parameter, adjusted geometric means		
	*C*_max_, ng/mL (90% CI)	AUC_tau_, h · ng/mL (90% CI)	*C*_12_, ng/mL (90% CI)
Effect of DRV/c on PK of temsavir (cohort 1)^*b*^			
Fostemsavir	2,045 (1,744–2,398)	101,214 (8,830–11,815)	219 (175–273)
Fostemsavir + DRV + COBI	3,665 (3,158–4,254)	20,092 (17,233–23,426)	490 (395–609)
GMR for fostemsavir + COBI + DRV vs fostemsavir	1.792 (1.619–1.983)	1.967 (1.778–2.176)	2.242 (1.746–2.878)
Effect of COBI on PK of temsavir (cohort 2)			
Fostemsavir	2,096 (1,890–2,324)	10,417 (9,105–11,919)	202 (156–261)
Fostemsavir + COBI	3,583 (3,007–4,269)	20,063 (16,918–23,791)	477 (364–625)
GMR for fostemsavir vs fostemsavir + COBI	1.709 (1.540–1.896)	1.926 (1.754–2.115)	2.363 (2.033–2.746)

a AUC_tau_, area under the concentration-time curve in one dosing interval; *C*_12_, concentration at 12 h after the last dose; CI, confidence interval; *C*_max_, maximum observed plasma concentration; COBI, cobicistat; DRV, darunavir; GMR, geometric mean ratio; PK, pharmacokinetic.

b One participant in cohort 1 was excluded from PK analysis due to emesis within 2 × *T*_max_ on day 4.

**(iii) Effect of FTR coadministration on ETR PK.** In cohort 2, compared with ETR alone, coadministration with FTR resulted in a small increase in the ETR *C*_max_, AUC_tau_, and *C*_12_ by 11%, 11%, and 14%, respectively. In cohort 3, compared with DRV/r plus ETR, coadministration with FTR increased the ETR *C*_max_, AUC_tau_, and *C*_12_ by 18%, 28%, and 28%, respectively. The 90% CIs of GMRs comparing FTR plus DRV/r plus ETR versus DRV/r plus ETR were contained within 0.75 to 1.70 (Table 3) for all three parameters in both cohorts.

### 206285 participant disposition and baseline demographics.

Of the 59 individuals who consented to participate, 32 entered the treatment period, with 16 participants each assigned to cohorts 1 and 2. Baseline demographics were generally balanced between treatment groups; the median age was 40 years (range, 25 to 50 years), 65.6% were male, 22 (68.8%) were white, and the median body weight was 80.85 kg (range, 51.3 to 104.7 kg).

### TMR PK in 206285.

**FIG 2:**
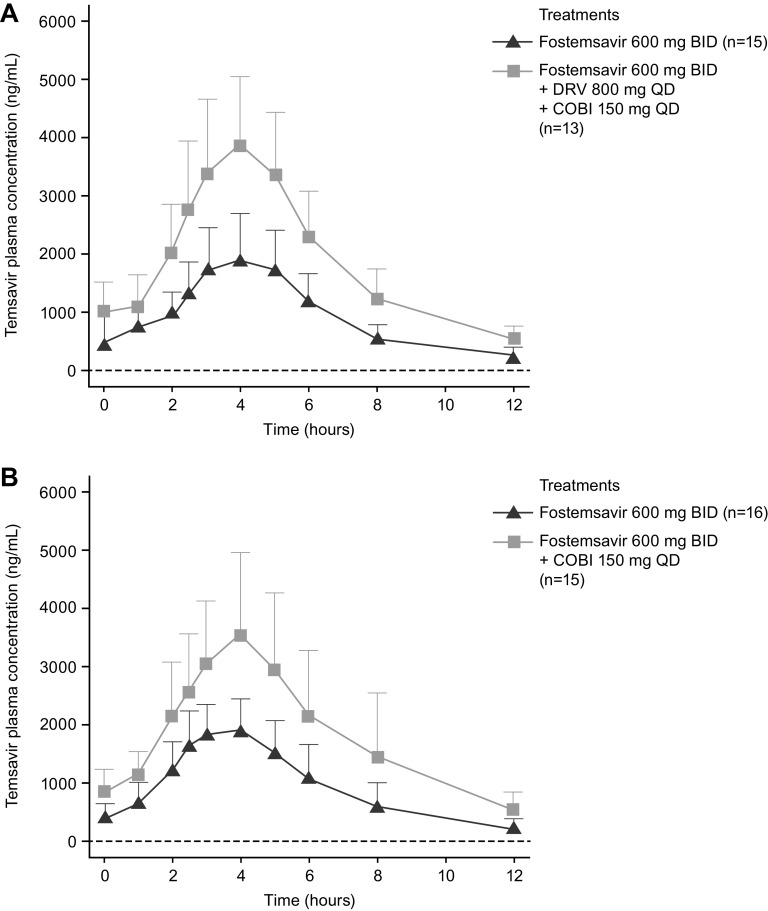
Effect of (A) DRV/c and (B) COBI alone on the PK parameters of temsavir. BID, twice daily; COBI, cobicistat; DRV, darunavir; PK, pharmacokinetics; QD, once daily.

**(i) Effect of DRV/c on TMR PK.** Compared with FTR alone, coadministration with DRV and COBI increased the TMR *C*_max_, AUC_tau_, and *C*_12_ by 79%, 97%, and 124%, respectively (Fig. 2A and Table 3). There was no clinically relevant change in the TMR *T*_max_ (median, min-max) when FTR was coadministered with DRV/c (4.00, 3.00 to 6.00 h) relative to administration of FTR alone (4.00, 2.50 to 5.00 h).

**(ii) Effect of COBI on TMR PK.** Compared with FTR alone, coadministration with COBI increased the TMR *C*_max_, AUC_tau_, and *C*_12_ by 71%, 93%, and 136%, respectively (Fig. 2B and Table 3). There was no clinically relevant change in the TMR *T*_max_ TMR (median, min-max) when FTR was coadministered with COBI (4.00, 3.00 to 5.00 h) relative to administration of FTR alone (3.00, 2.50 to 5.00 h).

**FIG 3:**
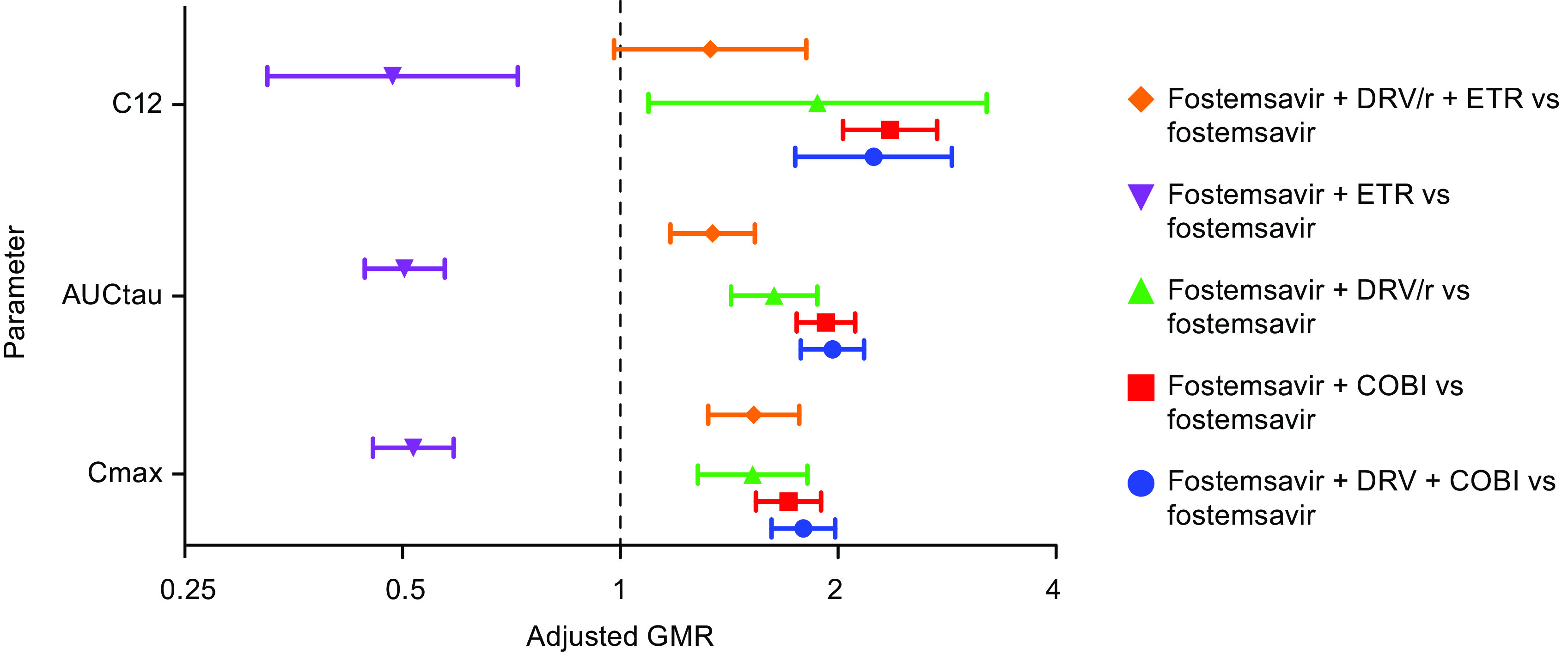
Effect of DRV plus COBI, COBI alone, DRV/r, ETR, and ETR plus DRV/r on the PK parameters of temsavir. Closed shapes represent adjusted GMRs, and connected bars represent 90% CIs of the adjusted GMRs. AUC_tau_, area under the concentration-time curve in one dosing interval; *C*_12_, concentration at 12 h after the last dose; CI, confidence interval; *C*_max_, maximum observed plasma concentration; COBI, cobicistat; DRV, darunavir; DRV/r, ritonavir-boosted DRV; ETR, etravirine; GMR, geometric mean ratio; PK, pharmacokinetics.

A forest plot representing the effect of DRV plus COBI, COBI alone, DRV/r, ETR alone, and ETR plus DRV/r on the PK parameters of TMR is shown in Fig. 3.

### Safety.

**(i) Study 206281.** Seven of the 46 (15.2%) participants randomized to study 206281 did not complete the study due to AEs, including 2 of 14 in cohort 1 and 5 of 18 in cohort 3. Reasons for discontinuation included mild or moderate skin rash that occurred 9, 11, or 12 days after the last dose of FTR in period 1 (days 13, 15, or 16, respectively) during either DRV/r administration in one participant or DRV/r plus ETR administration in four participants. Other reasons for discontinuation were back pain that occurred 4 days after the last dose of FTR in period 1 (day 8 during DRV/r plus ETR administration in one participant) and increased alanine aminotransferase that occurred during dosing of FTR with DRV/r and ETR (3 to 4 times the upper limit of normal; day 18 in one participant). There were no serious AEs (SAEs) or AEs categorized as severe or potentially life-threatening, and there were no deaths throughout the study.

A total of 22 participants (47.8%) reported AEs, with 50% of AEs reported in participants receiving DRV/r in combination with ETR (Table 4). AEs considered related to any of the study drugs were reported in 19 participants (41.3%). The most frequently reported drug-related AEs were skin and subcutaneous tissue disorders, including drug eruption in 13 participants (28.2%), generalized pruritus in 5 (10.8%), pruritus in 4 (8.7%), and dermatitis in 1 (2.2%). Drug-related headache was reported in six participants (13.0%). All AEs were mild to moderate in intensity. Four participants had posttreatment laboratory observations that met predefined criteria for a marked abnormality, including low absolute lymphocytes (*n* = 2), high absolute eosinophils (*n* = 1), high aspartate aminotransferase (*n* = 1), and high alanine aminotransferase (*n* = 1). No other notable clinical laboratory, electrocardiogram (ECG), vital sign, or physical examination results were observed.

**TABLE 4:** Summary of adverse events experienced by ≥2 participants in study 206281^*a*^

AE	No. of patients experiencing AE with treatments received (%)^*b*^							
	A (*n* = 46)	B (*n* = 14)	A + B (*n* = 13)	C (*n* = 14)	A + C (*n* = 14)	B + C (*n* = 18)	A + B + C (*n* = 13)	Total (*n* = 46)
Subjects with any AE	6 (13.0)	4 (28.6)	3 (23.1)	4 (28.6)	2 (14.3)	9 (50.0)	4 (30.8)	22 (47.8)
Drug eruption	0	3 (21.4)	0	2 (14.3)	0	7 (38.9)	1 (7.7)	13 (28.3)
Headache	1 (2.2)	1 (7.1)	1 (7.7)	0	0	3 (16.7)	1 (7.7)	6 (13.0)
Pruritus	1 (2.2)	1 (7.1)	1 (7.7)	0	0	0	3 (23.1)	6 (13.0)
Pruritus generalized	0	1 (7.1)	0	1 (7.1)	0	3 (16.7)	0	5 (10.9)
Back pain	0	1 (7.1)	0	0	0	2 (11.1)	0	3 (6.5)
Nausea	1 (2.2)	1 (7.1)	0	0	0	1 (5.6)	0	3 (6.5)
Abdominal distension	0	0	0	0	0	1 (5.6)	1 (7.7)	2 (4.3)
Aphthous stomatitis	0	0	0	0	0	1 (5.6)	1 (7.7)	2 (4.3)
Diarrhea	0	0	0	0	0	2 (11.1)	1 (7.7)	2 (4.3)
Dizziness	0	0	0	0	0	2 (11.1)	0	2 (4.3)
Dry mouth	1 (2.2)	0	0	0	0	1 (5.6)	0	2 (4.3)
Fatigue	0	1 (7.1)	0	0	0	1 (5.6)	0	2 (4.3)
Muscle spasms	0	0	0	0	0	2 (11.1)	0	2 (4.3)
Somnolence	0	0	0	0	0	2 (11.1)	0	2 (4.3)

a AE, adverse event; BID, twice daily; DRV/r, ritonavir-boosted darunavir; ETR, etravirine.

b Cohort 1 = A, B, A + B; cohort 2 = A, C, A + C; cohort 3 = A, B + C, A + B + C. A, fostemsavir 600 mg BID; B, DRV/r 600/100 mg BID; C, ETR 200 mg BID.

### Study 206285.

Of the 32 participants, 4 (12.5%) did not complete the study due to AEs, 3 (of 16) in cohort 1 and 1 (of 16) in cohort 2. In cohort 1, one participant who received FTR discontinued treatment on day 4 due to mild emesis, and two participants who received FTR coadministered with DRV/c discontinued treatment due to a moderate drug eruption on day 13 and mild emesis on day 8. In cohort 2, one participant discontinued due to mild pyrexia on day 6. All events, except pyrexia, were considered study drug-related. No deaths or SAEs were reported.

A total of 15 (46.9%) participants reported AEs, with the majority (37.5%) reported in participants receiving FTR with COBI in cohort 2 (Table 5). Most AEs were of mild or moderate intensity, and all except two were considered related to the study drug. AEs severe in intensity were reported in two participants, one after administration of FTR (headache) and the other after administration of FTR plus COBI (decreased neutrophil and white blood cell counts). No clinically significant findings in ECGs or vital signs/physical examinations were noted. An abnormal laboratory value of increased creatinine that did not meet marked laboratory abnormality criteria was associated with an AE in one participant (increased to 141 μmol/L and returned to within normal levels of 106 μmol/L by day 10); however, this did not result in discontinuation of any of the study drugs and resolved without sequelae.

**TABLE 5:** Summary of adverse events experienced by ≥2 participants in study 206285^*a*^

AE	No. of patients experiencing AE (%)				
	Cohort 1		Cohort 2		Total (*n* = 32)
	Fostemsavir (*n* = 16)	Fostemsavir + DRV + COBI (*n* = 15)	Fostemsavir (*n* = 16)	Fostemsavir + COBI (*n* = 16)	
Participants with AE	6 (37.5)	4 (26.7)	3 (18.8)	6 (37.5)	15 (46.9)
Headache	3 (18.8)	1 (6.7)	1 (6.3)	2 (12.5)	6 (18.8)
Neutrophil count decreased	0	0	0	2 (12.5)	2 (6.3)
WBC decreased	0	0	0	2 (12.5)	2 (6.3)
Back pain	1 (6.3)	0	1 (6.3)	0	1 (3.1)
Myalgia	0	2 (13.3)	0	0	2 (6.3)
Constipation	0	0	0	2 (12.5)	2 (6.3)
Nausea	1 (6.3)	1 (6.7)	0	0	2 (6.3)
Vomiting	1 (6.3)	1 (6.7)	0	0	2 (6.3)
Cough	0	0	0	2 (12.5)	2 (6.3)
Oropharyngeal pain	2 (12.5)	0	0	0	2 (6.3)
Pain	0	1 (6.7)	0	1 (6.3)	2 (6.3)

a AE, adverse event; COBI, cobicistat; DRV, darunavir; WBC, white blood count.

## DISCUSSION

TMR is a P-gp and BCRP substrate, and its metabolism is primarily mediated by esterase and CYP3A4 enzymes. TMR exposures increased by 71 to 136% when FTR was coadministered with the PK enhancer COBI, which inhibits CYP3A4, P-gp, BCRP, and other transporters. As increases in TMR exposure were similar between FTR plus COBI and FTR plus DRV/c, this suggests that the interaction is primarily driven by COBI, with no meaningful contribution from DRV. The coadministration of DRV/r with FTR resulted in a 52 to 88% increase in TMR exposures relative to administration of FTR alone. These findings are similar to those observed previously, where RTV alone increased TMR exposures by 44 to 53% (7), suggesting the observed interaction with DRV/r is primarily driven by the CYP3A and P-gp inhibitor, RTV. In the same study, another boosted PI, ATV/r, did not increase TMR exposures to a meaningful extent beyond what was observed with RTV alone (7). Taken together, TMR concentrations increase when FTR is given concomitantly with CYP3A4, P-gp, and/or BCRP inhibitors; the impact on TMR exposures is driven by the PK enhancer (RTV or COBI) when administered with the PI.

The impact of COBI (alone and as DRV/c) on TMR exposures was slightly higher than with DRV/r; however, as these drug interactions were assessed in different studies, it is difficult to attribute these differences entirely to the known drug interaction profiles of COBI or RTV. It is known that RTV induces several metabolic pathways, including CYP3A4 (*in vitro*), CYP1A2, CYP2C9, CYP2C19, and glucuronidation, which contributes to the different impact of RTV compared with COBI for certain drugs metabolized by these enzymes (14, 17). CYP3A4 contributes to the disposition of TMR, but not CYP1A2, CYP2C9, CYP2C19, or CYP2B6. Uridine diphosphate glucuronosyltransferase (UGT)-mediated metabolism is a minor clearance pathway for TMR (ViiV Healthcare, unpublished data), and induction of UGT by RTV, which likely does not occur with COBI, could contribute to the relatively smaller impact observed with RTV. In addition, the impact of BCRP inhibition by COBI on TMR may also contribute to these differences between COBI and RTV.

Coadministration of FTR with ETR resulted in an approximately 50% decrease in TMR exposures, consistent with induction of CYP3A by ETR. While the net impact of DRV/r and ETR when coadministered with FTR was an increase in TMR exposures, the increase was less than that observed with DRV/r without ETR, suggesting that ETR attenuates some inhibition of CYP3A by RTV. A similar phenomenon was observed with maraviroc, another ARV agent metabolized by CYP3A (18), where the impact of DRV/r plus ETR on the increase in maraviroc exposure was less than that of DRV/r without ETR. The reduced TMR exposure when FTR is given in combination with ETR is not expected to impact FTR efficacy. A model-based assessment of the relationship between TMR exposures (*C*_tau_ adjusted for the protein-binding adjusted 50% inhibitory concentration [IC_50_]) and viral load decline following 8 days of monotherapy in HIV-1-infected participants demonstrated similar response across a wide range of FTR doses (400 mg twice a day [BID], 600 mg once a day [QD], 600 mg plus RTV every 12 h, 800 mg QD, 1,200 mg QD, and 1,200 mg every 12 h with and without RTV) (19). In addition, simulations showed that PK changes associated with effects of moderate CYP3A inducers, strong CYP3A inhibitors, prandial status, and extremes of body weight do not result in clinically relevant changes in the day 8 virologic response for FTR 600 mg BID, and no FTR dose adjustment is needed for these factors (20). The coadministration of FTR with DRV/r and/or ETR did not have a clinically relevant effect on exposures of DRV or ETR, as the 90% CIs were contained within the predefined boundaries of 0.75 to 1.70, consistent with a lack of effect of FTR and TMR on the metabolic pathways of ETR (methyl hydroxylation, with subsequent glucuronidation of the metabolites) and DRV (carbamate hydrolysis).

In these studies, administration of FTR alone or in combination with COBI, DRV/c, DRV/r, and/or ETR alone did not change the safety profile of FTR in healthy participants. Furthermore, the increased exposures to TMR observed when FTR was coadministered with COBI, DRV/c, DRV/r, or DRV/r plus ETR do not impact the safety profile of FTR in HIV-1-infected participants (19). An exposure-response assessment of safety endpoints of interest, including AEs and laboratory parameters in HIV-1-infected participants administered FTR across a wide range of doses as monotherapy for 8 days as well as in combination with raltegravir and tenofovir disoproxil fumarate for 24 weeks, demonstrated no apparent relationship with TMR concentrations (19). TMR *C*_max_-driven QT prolongation was observed at a supratherapeutic dose of FTR (2,400 mg BID) (21, 22); however, this QT prolongation occurred at exposures that exceed the geometric mean exposures of TMR in the current set of studies by >2-fold.

Collectively, results from these studies in conjunction with previously reported data support no dose adjustment of FTR when coadministered with strong CYP3A inhibitors, P-gp or BCRP inhibitors, and modest inducers, including PK enhancer-containing regimens and regimens that contain ETR, nevirapine, and efavirenz based on the therapeutic margin for TMR. These data also informed the design of the phase 3 study of FTR in an HTE HIV-1-infected patient population and provided important information for the coadministration of FTR with commonly prescribed ARVs.

## MATERIALS AND METHODS

### Study designs.

Study 206281 (ClinicalTrials.gov registration no. NCT02063360) was an open-label, single-sequence, multiple-dose, three-cohort study. Participants were randomly assigned to one of three cohorts per a computer-generated randomization scheme.

**FIG 4:**
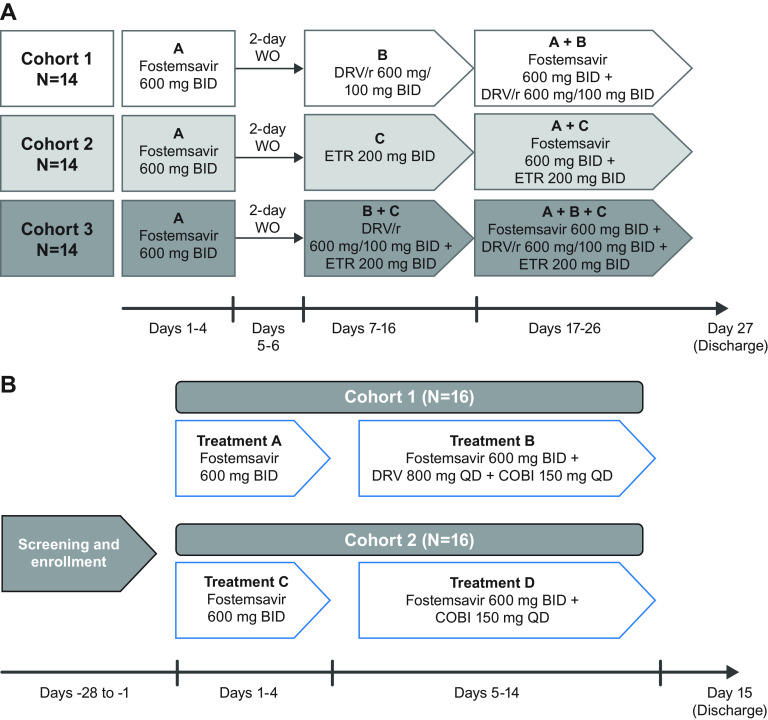
Study designs for (A) 206281 and (B) 206285. BID, twice daily; COBI, cobicistat; DRV, darunavir; DRV/r, ritonavir-boosted darunavir; ETR, etravirine; WO, washout.

• Cohort 1: Participants received FTR at 600 mg twice daily (BID) on days 1 to 4. After a 2-day washout (days 5 and 6), participants received DRV/r at 600/100 mg BID for 10 days (days 7 to 16) followed by FTR at 600 mg BID with DRV/r at 600/100 mg BID for 10 days (days 17 to 26).
• Cohort 2: Participants received FTR at 600 mg BID on days 1 to 4. After a 2-day washout (days 5 and 6), participants received ETR at 200 mg BID for 10 days (days 7 to 16) followed by FTR at 600 mg BID with ETR at 200 mg BID for 10 days (days 17 to 26).
• Cohort 3: Participants received FTR at 600 mg BID on days 1 to 4. After a 2-day washout (days 5 and 6), participants received DRV/r at 600/100 mg BID and ETR at 200 mg BID for 10 days (days 7 to 16) followed by FTR at 600 mg BID with DRV/r at 600/100 mg BID and ETR at 200 mg BID for 10 days (days 17 to 26) (Fig. 4A).

Study 206285 (ClinicalTrials.gov registration no. NCT02277600) was an open-label, single-sequence, multiple-dose, two-cohort, one-way interaction study. Participants were sequentially enrolled to two cohorts.

• Cohort 1: Participants received FTR at 600 mg BID on days 1 to 4. Participants then received FTR at 600 mg BID plus DRV at 800 mg and COBI at 150 mg once daily (QD) on days 5 to 14.
• Cohort 2: Participants received FTR at 600 mg BID on days 1 to 4. Participants then received FTR at 600 mg BID plus COBI at 150 mg QD on days 5 to 14 (Fig. 4B).

Eligible participants were admitted to the clinical facility on day −1 of each study. Study 206281 and 206285 participants were discharged on day 27 and day 15, respectively. For study 206281, predose trough blood samples for PK analysis of TMR were collected on days 3, 24, and 25, and serial blood samples for PK analysis of TMR were collected on days 4 and 26 in all cohorts. Predose trough blood samples for PK analysis of DRV/r were collected on days 14, 15, 24, and 25, and serial blood samples for PK analysis of DRV/r were collected on days 16 and 26 for DRV/r (cohorts 1 and 3) and ETR (cohorts 2 and 3). For study 206285, blood samples were collected for up to 12 h after the morning study drug administration on days 4 and 14 for TMR PK analysis, and for up to 24 h after the morning study drug administration on day 14 for COBI PK analysis. Physical examinations, measurements of vital signs, 12-lead ECGs, and clinical laboratory evaluations were performed, and participants were closely monitored for AEs and SAEs, throughout both studies.

Selection of the 600-mg FTR dose was based on phase 2 results using PK/pharmacodynamic (PD) modeling and computational predictions (19) and is the approved dose of FTR. DRV/c, DRV/r, COBI, and ETR were administered at standard oral doses (23). All doses of study drug were administered with standard meals.

### Participants.

Both studies were conducted in healthy male and female participants, as determined by not having any clinically significant findings following medical history review, physical examination, 12-lead ECG measurements, and clinical laboratory test results. Participants were 18 to 50 years old (inclusive), with a body mass index of 18 to 32 kg/m^2^. Individuals were excluded from participation if they were pregnant or breastfeeding. Written informed consent was obtained from all participants. Both studies were conducted in accordance with good clinical practice, as defined by the International Conference on Harmonisation, and in accordance with the ethical principles underlying European Union Directive 2001/20/EC and the U.S. Code of Federal Regulations (CFR), Title 21, Part 50 (21CFR50).

### Study objectives.

The primary objective of study 206281 was to assess the effects of DRV/r, ETR, and DRV/r plus ETR on the PK of TMR in healthy participants. Secondary objectives included the assessment of the effect of FTR on the PK of RTV, DRV, and ETR, and assessment of the safety and tolerability of FTR administered alone and in combination with the other study drugs.

The primary objective of study 206285 was to assess the effects of DRV/c and COBI alone on the PK of TMR in healthy participants. Secondary objectives included the assessment of TMR steady-state PK with and without the coadministration of DRV/c and COBI alone and assessment of the safety and tolerability of FTR alone and in combination with the other study drugs.

### Assessments.

**(i) Bioanalytical methods and PK analysis.** Following extraction from dipotassium ethylenediaminetetraacetic acid (K_2_EDTA)-anticoagulated plasma, TMR, DRV, COBI, RTV, and ETR concentrations were measured using validated liquid chromatography-tandem mass spectrometry assays. The lower limit of quantification and the upper limit of quantification for the analysis of TMR in both studies were 5 ng/mL and 5,000 ng/mL, respectively. For the few TMR samples over the calibration range, an aliquot factor was used, and the samples were reassayed. Those for the analysis of COBI were 5 ng/mL and 5,000 ng/mL, respectively; those for DRV and RTV were 10 ng/mL and 10,000 ng/mL, respectively, and those for ETR were 0.5 ng/mL and 500 ng/mL, respectively. The within-run and between-run precisions for quantification of TMR in study 206281 were ≤5.49% and ≤3.87%, respectively. Those for the quantification of DRV were ≤9.22% and ≤5.49%, respectively; those for COBI were ≤5.4% and ≤7.1%, respectively; those for RTV were ≤8.14% and ≤3.05%, respectively; and those for ETR were ≤15.1% and ≤7.13, respectively. The within-run and between-run precisions for quantification of TMR in study 206285 were ≤7.68% and ≤5.30%, respectively.

**(ii) PK analysis.** For both studies, individual participant PK parameter values were derived by noncompartmental methods with a validated PK analysis program using actual times. The PK parameter *C*_max_, *T*_max_, AUC_tau_, and *C*_12_ were reported for TMR, RTV, and DRV (206281 only; samples for ETR and DRV PK were not collected in 206285). The PK parameters *C*_max_, *T*_max_, AUC_tau_, and concentration at 24 h after the last dose (*C*_24_) were determined for COBI (the COBI PK was consistent with published data; thus, the interactions were assessed at clinically relevant concentrations [14]). *C*_max_, *T*_max_, *C*_12_, and *C*_24_ were recorded directly from experimental observations and AUC_tau_ was calculated by mixed log-linear trapezoidal summations.

### Statistical analysis.

SAS software version 9.2 (SAS Institute, Inc., Cary, North Carolina) was used for all statistical analyses, tabulations, and graphical presentations. For all treatment comparisons, a linear mixed-effect model was fitted to the log-transformed data with treatment as fixed effect and measurements within each participant as repeated measures, for use in the estimation of effects and the construction of CIs. Point estimates and 90% CIs for differences on the log scale were exponentiated to obtain estimates for GMRs and respective 90% CIs on the original scale. For study 206281, the planned sample size was 12 participants with an 80% probability for the 90% CI to be within 82% and 122% of the point estimate of the GMR for the TMR *C*_max_ and within 87% and 115% of the point estimate of the GMR for the TMR AUC_tau_. For study 206285, the planned sample size was 13 participants per cohort with an 80% probability for the 90% CI to be within 83% and 120% of the point estimate of the GMR for the TMR *C*_max_ and within 88% and 113% of the point estimate of the GMR for the TMR AUC_tau_. FTR administered alone was the reference treatment for both studies. A range of 0.75 to 1.70 was the non-clinically significant 90% CI for DRV and ETR in study 206281 based on the prescribing information for these agents (12, 13).

### Data availability.

Anonymized individual participant data and study documents can be requested for further research from www.clinicalstudydatarequest.com.
