# Supplementary material for: Pharmacokinetics of Temsavir, the Active Moiety of the HIV-1 Attachment Inhibitor Prodrug, Fostemsavir, Coadministered with Cobicistat, Etravirine, Darunavir/Cobicistat, or Darunavir/Ritonavir with or without Etravirine in Healthy Participants
Source: Antimicrob Agents Chemother. 2022 Mar 22;66(4):e02251-21. doi: 10.1128/aac.02251-21 (PMC9017385; doi:10.1128/aac.02251-21)
Supplement: Supplemental file 1 — Tables S1 and S2. Download aac.02251-21-s0001.pdf, PDF file, 0.08 MB [file aac.02251-21-s0001.pdf]

TABLE S1 Subject disposition for 206281

	Cohort 1	Cohort 2	Cohort 3	Total
Number of participants enrolled, n (%)	—	—		100 (100)
Number of subjects entering treatment period, n (%)	14 (14)	14 (14)	18 (18)	46 (46)
Number of subjects not entering treatment period, n (%)	—	—	—	54 (54)
Subject withdrew consent				2 (2)
Subject no longer met study criteria				38 (38)
Other reasons				14 (14)
Number of subjects completing the study, n (%)	12 (12)	14 (14)	13 (13)	39 (39)
Number of subjects not completing the study, n (%)	2 (2)	0	5 (5)	7 (7)
Reason for not completing the study, n (%)				
Adverse event	2 (2)	0	5 (5)	7 (7)

TABLE S2 Subject disposition for 206285

	**Treatment AB**	**Treatment CD**	**Total**
Number of participants enrolled, n (%)	—	—	59
Number of subjects entering treatment period, n (%)	16	16	32 (54.2)
Number of subjects not entering treatment period, n (%)	—	—	27 (45.8)
Subject withdrew consent			5 (8.5)
Lost to follow-up			3 (5.1)
Subject no longer met study criteria			19 (32.2)
Number of subjects completing the study, n (%)	13 (81.3)	15 (93.8)	28 (87.5)
Number of subjects not completing the study, n (%)	3 (18.8)	1 (6.3)	4 (12.5)
Reason for not completing the study, n (%)			
Adverse event	3 (18.8)	1 (6.3)	4 (12.5)
